# Fabrication of cellulose nanocrystals/carboxymethyl cellulose/zeolite membranes for methylene blue dye removal: understanding factors, adsorption kinetics, and thermodynamic isotherms

**DOI:** 10.3389/fchem.2024.1330810

**Published:** 2024-02-02

**Authors:** Mostafa Ahmed Ibrahim, Ahmed Salama, Fouad Zahran, Mohamed Saleh Abdelfattah, Ali Alsalme, Mikhael Bechelany, Ahmed Barhoum

**Affiliations:** ^1^ NanoStruc Research Group, Chemistry Department, Faculty of Science, Helwan University, Cairo, Egypt; ^2^ Production and R&D Unit, NanoFab Technology Company, Giza, Egypt; ^3^ Cellulose and Paper Department, National Research Centre, Giza, Egypt; ^4^ Chemistry Department, Faculty of Science, Helwan University, Cairo, Egypt; ^5^ Marine Natural Products Research Unit, Faculty of Science, Helwan University, Cairo, Egypt; ^6^ Department of Chemistry, College of Science, King Saud University, Riyadh, Saudi Arabia; ^7^ Institut Européen des Membranes (IEM), UMR 5635, University of Montpellier, ENSCM, CNRS, Montpellier, France; ^8^ Gulf University for Science and Technology, GUST, Kuwait

**Keywords:** carbohydrate polymers, cross-linked membranes, nanocomposites, organic pollutants, methylene blue, adsorption parameters

## Abstract

This study introduces environmentally-friendly nanocellulose-based membranes for AZO dye (methylene blue, MB) removal from wastewater. These membranes, made of cellulose nanocrystals (CNCs), carboxymethyl cellulose (CMC), zeolite, and citric acid, aim to offer eco-friendly water treatment solutions. CNCs, obtained from sugarcane bagasse, act as the foundational material for the membranes. The study aims to investigate both the composition of the membranes (CMC/CNC/zeolite/citric acid) and the critical adsorption factors (initial MB concentration, contact time, temperature, and pH) that impact the removal of the dye. After systematic experimentation, the optimal membrane composition is identified as 60% CNC, 15% CMC, 20% zeolites, and 5% citric acid. This composition achieved a 79.9% dye removal efficiency and a 38.3 mg/g adsorption capacity at pH 7. The optimized membrane exhibited enhanced MB dye removal under specific conditions, including a 50 mg adsorbent mass, 50 ppm dye concentration, 50 mL solution volume, 120-min contact time, and a temperature of 25°C. Increasing pH from neutral to alkaline enhances MB dye removal efficiency from 79.9% to 94.5%, with the adsorption capacity rising from 38.3 mg/g to 76.5 mg/g. The study extended to study the MB adsorption mechanisms, revealing the chemisorption of MB dye with pseudo-second-order kinetics. Chemical thermodynamic experiments determine the Freundlich isotherm as the apt model for MB dye adsorption on the membrane surface. In conclusion, this study successfully develops nanocellulose-based membranes for efficient AZO dye removal, contributing to sustainable water treatment technologies and environmental preservation efforts.

## Graphical Abstract

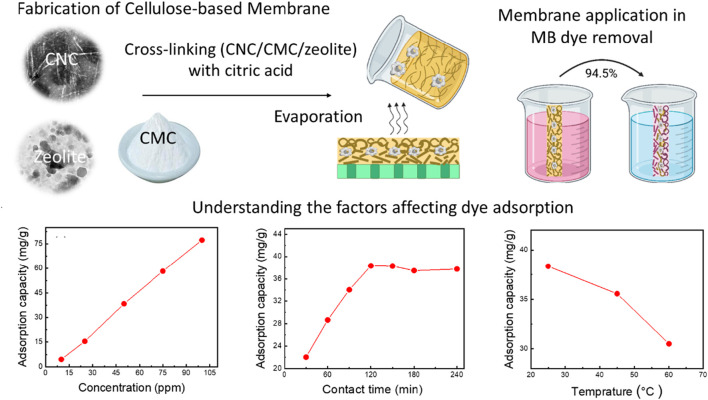

## 1 Introduction

Clean water is one of the most crucial requirements for the survival of all things that are alive. However, rapid industrialization in the modern era has led to widespread water pollution, posing a significant environmental threat to human health and ecological systems (Nnaji et al., 2018). Water pollutants can be classified into three main categories: organic compounds as soaps, dyes, pharmaceutical compounds, fertilisers, pesticides, petrochemicals, and oils, inorganic compounds as heavy metals (Sharma et al., 2018a) and radioactive substances (Sharma et al., 2017a), and microorganisms as viruses, bacteria, fungi. Organic dyes are among the most dangerous pollutants (Salama et al., 2021). They are usually not biodegradable and harmful even in small amounts (L ppm). Textile wastewater typically contains toxic dyes at concentrations of 10–200 ppm, in addition to various organic and inorganic contaminants (Liu et al., 2021; Umapathi et al., 2022; Raju et al., 2023). Even after treating the wastewater from dye baths, approximately 90% of these dyes enter river systems without undergoing chemical changes. These dyes pose a significant threat to aquatic plants and animals, accumulating in water bodies and hindering sunlight penetration into the depths. This interference impairs the ability of plants to carry out photosynthesis (Jamee and Siddique, 2019). Dyes also lower the oxygen levels in the water, leading to the death of aquatic animals and plants. Moreover, they can inhibit plant growth, enter the food chain, reach human organs, and promote cytotoxicity, mutagenicity, and carcinogenicity (Gherbi et al., 2022).

Methylene blue (MB) commonly used dye in various industrial processes, poses severe environmental threats due to its persistent and harmful characteristics. As an organic dye, MB’s resistance to biodegradation makes it highly persistent in water ecosystems, particularly in wastewater from textile industries. MB dye removal methods can be accomplished by physical, chemical, and biological techniques. It was reported that adsorption is a high-quality treatment process for removing dyes from industrial wastewater (Barhoum et al., 2021). The driving force for adsorption is unsaturated forces (hydrogen bonding, dipolar forces, and non-specific hydrophobic interactions) at the adsorbent, which can form bonds with the adsorbate (Hamimed et al., 2022). Batch adsorption studies were conducted to investigate the effects of adsorbent mass, initial concentration, temperature, contact time, pH, and ionic strength of the dye solution. The MB dye was absorbed more as the contact time, temperature, amount of adsorbent, and initial concentration increased (Laouini et al., 2021). Various adsorbents such as carbon materials (carbon black, graphene, carbon nanotubes), inorganic oxides (Ag/Ag_3_PO_4_, Fe_2_O_3_, TiO_2_, ZnO, SiO_2_, zeolite) (Rehan et al., 2019), polymers (cellulose derivatives, chitosan, polyacrylamide) (Meftahi et al., 2022a), and microorganisms (bacteria, microalgae, and fungi) have proven effective in degrading and removing MB dye from wastewater (Barhoum et al., 2017). Naturally occurring adsorbents, specifically cellulose-based membranes, are extensively researched for dye removal due to their environmentally friendly nature, non-toxic properties, and ease of modification (Bouafia et al., 2022).

Cellulose nanomaterials (nanofibers, nanocrystals, nanoparticles) are used in various wastewater treatment applications (Sjahro et al., 2021; Chen et al., 2022). Cellulose nanocrystals (CNCs) are commonly extracted from cellulose sources, including a diverse range of woods such as pine, birch, oak, and Cassava (Egbosiuba, 2022), as well as from other biological materials like bacteria, algae, tunicates, and more (Sharma et al., 2019; Jeevanandam et al., 2021). Agricultural wastes (e.g., rice straw and bagasse) are also potential raw materials for the production of CNCs (Jeevanandam et al., 2021; Thakur et al., 2020). Alkali treatment with NaOH and acid hydrolysis with HCl or H_2_SO_4_ are two common methods for producing CNCs from agricultural wastes. Alkali treatment typically is used to remove lignin, hemicellulose, and other impurities. Acid hydrolysis is used to break down the cellulose microfibers into CNCs and form negatively charged groups that electrostatically stabilize the CNCs (Barhoum et al., 2020). In addition, the negatively charged groups (-OH, -SO_3_, -COO) on the CNCs exert an electrostatic attraction on heavy metals and cationic dyes (Meftahi et al., 2022b). The CNCs can also be chemically modified to increase their affinity for anionic and non-ionic dyes. Novel adsorbents based on CNCs for the removal of dyes can be obtained by: 1) graft polymerization of CNCs with functional molecules; 2) combination with other cellulose derivatives (CMC); 3) combining with inorganic adsorbents (e.g., clay, zeolite) (Kovo et al., 2023); 4) combining with carbon nanomaterials (carbon black, graphene, carbon nanotubes) (Gomri et al., 2022).

This study introduces an innovative nanocellulose-based membrane, incorporating CNC, CMC, zeolites, and citric acid, for the targeted removal of MB dye from wastewater. A substantial percentage of these dyes, approximately 15%–20%, are discharged into water systems without proper treatment. The use of CNC sourced from sugarcane bagasse and natural zeolites with well-defined nanopores aligns with eco-friendly practices (Mohammed et al., 2021). The membrane, enriched with active groups such as carboxylate, hydroxyl, and sulfate ester, exhibits heightened MB adsorption capacity. Zeolite inclusion enhances pore structure efficiency for improved adsorption and ion exchange capabilities. Citric acid, serving as a cross-linking agent, not only reinforces structural integrity but also forms complexes with MB dye, influencing adsorption behaviour (Shen et al., 2015). Rigorous exploration of the membrane’s composition, finely tuned adsorption parameters, and application of mathematical models provide a detailed understanding of the chemical kinetics and thermodynamics involved in dye removal. This study significantly contributes to advancing sustainable water treatment approaches, emphasizing the potential of nanocellulose-based membranes to overcome environmental challenges linked to dye pollution.

## 2 Experimental

### 2.1 Materials

Sugarcane bagasse was derived from plantations located in Cairo, Egypt. Hydrochloric acid (HCl, 37%, Merck), sodium hydroxide (NaOH, 98%, Daejung, Siheung-si, Gyeonggi-di, Korea), hydrogen peroxide (H_2_O_2_, 52%, Diachem chemicals, Inc., United States), sodium hypochlorite (NaOCl, 13%, Chem-Lab NV, Belgium), and sulfuric acid (H_2_SO_4_, 97%, Emparta, Germany) were used for the synthesis of CNC. Carboxymethyl cellulose sodium salt (C_8_H_16_O_8_, medium viscosity, Loba Chemie, India), Methanol (CH_3_OH, CDH company, New Delhi, India), nitric acid (HNO₃, Piochem company, Giza, Egypt) methylene blue (C_16_H_18_ClN_3_S, El-Nasr Company, Cairo, Egypt), citric acid (C₆H₈O₇, 99%, Sigma-Aldrich), and zeolite (Clinoptilolite., (Na, K, Ca)_2-3_A_l3_(Al, Si)_2_Si_13_O_36_.12H_2_O, 99%, Nano Fab Technology, Egypt). During the experiments, MilliQ water having a resistivity of 18.2 million ohm-cm (18.2 megohms) and conductivity of 0.055 microsiemens was used.

### 2.2 Synthesis of CNC from bagasse

Synthesis of CNC was carried out in seven successive steps. Firstly, sugarcane bagasse was sun-dried for 24 h and subsequently cut into small pieces measuring 1 cm × 1 cm. A quantity of 20 g of dried sugarcane bagasse was subjected to dewaxing using 1,000 mL of water, heated to 90°C for 2 h with continuous stirring, and the process was repeated twice to ensure the complete removal of waxy substances (Mzimela et al., 2018). Secondly, the dewaxed powder was treated with 400 mL of 10v/v% HCl at 60°C for 2 h to eliminate inorganic minerals. Thirdly, the demineralized powder was thoroughly washed with water until a neutral pH was achieved, ensuring that any remaining acid and minerals were effectively removed. Following this, the resulting pulp was subjected to alkalization with 350 mL of 4 w/v % NaOH, with continuous stirring at 60°C for 2 h to remove lignin and hemicellulose. The pulp was again washed with distilled water until it reached a neutral pH. In the fifth step, the pulp underwent bleaching with 250 mL of 24v/v% H_2_O_2_ while being mechanically agitated at 60°C for 2 h, and then it was washed until a neutral pH was attained. In the sixth step, the pulp was bleached with 200 mL of 2 wt/v% NaOCl under mechanical stirring at 90°C for 4 h. Subsequently, it was washed, filtered, and dried to yield chemically purified pulp. Finally, CNC was obtained through acid hydrolysis of the cellulose fibers obtained in the previous step. This involved treating the fibers with 65 v/v% H_2_SO_4_ for 2 h at 45°C with vigorous stirring. The resultant CNC was rinsed multiple times until the solution reached a neutral pH, then centrifuged at 4,000 rpm for 15 min before being dried overnight at 50°C (Das et al., 2022).

### 2.3 Determination of degree of substitution of carboxymethyl cellulose

A standard procedure (ASTM 1961) was used to check the values for the degree of substitution (DS) (Toğrul and Arslan, 2003). Briefly, 21.6 mL of 65% HNO_3_ and 178.4 mL of 90% methanol were mixed together and then 5 g CMC was dispersed in the mixture and allowed to stand for 3 h under continuous stirring. The CMC was filtrated and the excess acid was then washed out with 70% methanol. The CMC powder was then allowed to dry at 55°C for 1 h. Next, 200 mL of distilled water and 30 mL of 1 M NaOH were used to dissolve a 2 g portion of Na-CMC. The mixture was dissolved and titrated with 1N HCl (Labet and Thielemans, 2012). The following formulas (Eqs 1, 2) were used to calculate the DS of CMC.

$$A=\left(\text{BC}-\text{DE}\right)/F$$ (1)

$$\text{DS}=0.162A/\left(1-0.58A\right)$$ (2)

(K et al., 1997) where A is the equivalent weight of alkali required per gram of sample; B (mL), is the volume of NaOH solution; C (N); is the normality of NaOH solution; D (mL), is the volume of HCl solution; E (N); is the normality of HCl solution; F (g) is the weight of the sample (CNC in dry powder form). The volume used after the titration of HCL = 24 mL, so A = 3 and DS can be calculated from the equation.

### 2.4 Membrane fabrication and characterization

Six membranes were prepared with different dry weight concentrations of 0–60 wt% CNC, 15–75 wt% CMC, 0–20 wt% zeolite, and 5 wt% citric acid (see Table 1). The solid content of the membrane (CNC, CMC, zeolite, citric acid) was adjusted to 1 g in 20 mL of water. First, CNC, CMC, and zeolite were mixed ultrasonically for 30 min and then magnetically stirred for 60 min. The crosslinking agent (concentrated citric acid solution) was added and the mixture was stirred with a mechanical stirrer for 15 min. The mixture was then left overnight to remove air bubbles. The mixture was poured into Petri dishes (9 cm diameter) and air-dried for 48 h. The dried membrane was dried at 60°C for 8 h to speed up the reaction between the citric acid and the other components (CNC, CMC, and zeolite).

**TABLE 1:** Six membranes were prepared using different concentrations of CNC, CMC, zeolite, and citric acid.

Membranes	Membranes composition				Dye removal efficiency	
	CNC (%)	CMC (%)	Zeolite (%)	Citric (%)	Removal percentage (%)	Removal capacity
M0	0.0	75	20	5	65.4 ± 0.6	32.7 ± 0.9 mg/g
M1	47.5	47.5	0	5	67.4 ± 0.5	33.7 ± 0.7 mg/g
M2	37.5	37.5	20	5	68.6 ± 0.7	34.3 ± 0.5 mg/g
M3	50	25	20	5	71.2 ± 0.8	35.6 ± 0.8 mg/g
M4	37.5	18.5	20	5	72.5 ± 0.7	36.3 ± 0.6 mg/g
M5	60	15	20	5	76.5 ± 0.9	38.2 ± 0.9 mg/g

The adsorption experiments were conducted at 50 mg of the membrane, initial MB concentration of 50 ppm, solution pH = 7, room temperature of 25°C, and contact time of 2 h.

The physicochemical properties of the zeolite, CNC, and prepared membranes were characterized using different techniques. The crystalline phase of the CNC and zeolite was determined using an X-ray diffractometer measurements with Empyrean PANalytical diffractometer utilizing a Cu K α1 radiation (*λ* = 1.54056 Å) and step size of 0.05° at room temperature. For XRD analysis, the CNC (powder) and zeolite (powder) were milled using a planetary ball mill (LZQMO.4L, Shicheng Oasis Mineral Equipment Manufacturing Co., LTD.) for 0.5 h at 2,500 RPM, which did not affect the crystallinity of the samples (Mattonai et al., 2018). Fourier transform infrared spectrophotometer (FTIR, JASCO MODEL 6700, United States) was employed to investigate the changes in the functional groups of the CNC and CMC after citric acid crosslinking. FTIR spectra were recorded as KBr pellets (1wt% sample in anhydrous KBr) in the spectral range of 4,000–400 cm^−1^ with a resolution of 4 cm^−1^ and an accumulation of 64 scans. The particle size and morphology of the CNC and zeolite particles were examined by transmission electron microscope (TEM, JEOL-JEM -2100, Tokyo, Japan). The operating parameters were as fellow: accelerating potential of 100 keV, magnification range of ×6000, and pressure uses is 10^–4^ Pa. Field emission scanning electron microscopy (FE-SEM, EOL JSM-6510LV QSEM, Tokyo, Japan) was used to examine the surface morphology and elemental composition of the membranes. The operating conditions were as fellow: accelerating voltage of 15 kV, magnification range of ×800, horizontal field width of 259 μm, the working distance of 7.8 mm, and pressure of 50 Pa. Microtrac BELSORP was used to determine the BET surface area and pore size distribution. The swelling properties of the membrane were determined by immersing the membranes (1 cm × 1 cm) in 50 mL of water for 24 h at room temperature. The dry weight of the membrane was determined before and after water immersion, and the swelling percentage was calculated using formula (Eq. 3):

$$\left.\text{Swelling }\%=\left(W2/W2-W1\right)\times 100\right)$$ (3)

(Padaki et al., 2012) where W2 is the weight of the swollen sample after immersion and W1 is the dry weight of the membrane.

### 2.5 Adsorption studies

Removal of MB dye using the prepared membranes was performed in batch adsorption experiments using a UV-vis spectrophotometer (PerkinElmer Lambda 41) at *λ*_max_ = 665 nm (Steinfeld et al., 2015). The adsorption parameters include the mass of the membrane (50 mg), contact time (from 30 min to 240 min), initial dye concentrations (from 10 ppm to 100 ppm), pH (from 3 to 12), and temperatures (from 25°C, 45°C to 60°C) were studied. The volume of the solution dye was kept constant at 50 mL and the pH was adjusted using diluted NaOH and HCl. The equilibrium adsorption capacity Qe (mg/g) and percentage of MB dye removal (%) were calculated using Eqs 4, 5:

$$\text{Qe}=\left(C0\;-\text{ Ce}\right)\;V\;/\;W$$ (4)

$$\text{Dye removal }\left(\%\right)=\left(C0\;-\text{ Ce}\right)\;x\;100\;/\;C$$ (5)

(Egbosiuba et al., 2020) where Ce (mg/L or ppm) is the adsorbate equilibrium concentration and C_0_ (mg/L or ppm) is the initial MB concentration (Egbosiuba et al., 2020). The volume of the solution is V (L, 50 mL dye solution), and the weight of the adsorbent is W (g, 5 mg).

### 2.6 Point of zero charge

Point of zero charges (pHpzc) is the pH for which the net surface charge of the adsorbent is equal to zero (Luyen et al., 2021). The pHpzc was determined by a simple electrochemical method in which 50 mL of 0.05 M NaCl was added to a series of beakers, the pH was adjusted to precise values between 2 and 12 with 0.1 M HCl or 0.1 M NaOH, and then 40 mg of adsorbent was added to each beaker. The mixture was shaken continuously for 48 h at room temperature to obtain the final pH. The pHpzc is the point where the curve representing pH = (pH-final - pH-initial as a function of pH-initial intersects the abscissa axis.

### 2.7 Adsorption kinetics

Adsorption kinetics was utilized to determine the MB dye’s adsorption capacity as a function of contact time at 50 mg membrane mass, 50 ppm initial MB dye concentration, pH = 7, and 25°C ambient temperature. The experimental kinetic data were then fitted by the pseudo-first-order and pseudo-second-order as adsorption kinetic models. The linear form of the pseudo-first-order kinetic model of adsorption solid-liquid systems is given in Eq. 6:

$$\log\;\left(q_{e\;}-\;q_{t\;}\right)=\log\left(q_{e\;}\right)-\;\frac{K_{1}}{2.303}\;t$$ (6)

The pseudo-second-order kinetic model is based on the MB chemisorption hypothesis and is given by Eq. 7:

$$\frac{t}{q_{t}}=\frac{t}{q_{e}}+\frac{1}{K_{2}q_{e}^{2}}$$ (7)

(Uddin et al., 2017) where q_t_ (mg/g) represents the adsorption capacity at the given contact time t and q_e_ (mg/g) represents the adsorption capacity at the equilibrium time. The rate constants (k_1_ and k_2_) are for pseudo-first-order and pseudo-second-order, respectively (Uddin et al., 2017).

### 2.8 Thermodynamic isotherm

Adsorption thermodynamics was utilized to investigate the best membrane (M5) for MB dye adsorption in terms of favorability, reversibility, and energy. Langmuir isotherm and Freundlich isotherm were applied to understand the interactions between MB dye and the membrane (Basu et al., 2018). The Van’t Hoff equation Eqs 8, 9) was then used to estimate thermodynamic parameters such as a change in Gibbs free energy (∆G°), change in enthalpy (∆H°), and change in entropy (∆S°).

ln⁡K=−∆G°/R T (8)

$$\ln K=-\;\left(∆H/\text{RT}\right)+\left(∆S{}^{\circ}/R\right)$$ (9)

(Basu et al., 2018) in which K = Qe/Ce represents the linear adsorption distribution coefficient and also the so-called equilibrium constant, T represents the temperature (K) of the solution, and R represents the gas constant (8.314 J/mol·K). The change in enthalpy (∆H°), and change in entropy (∆S°) are calculated from the slope and intercept of the ln K vs. 1/T plot, respectively (Shenvi et al., 2015).

## 3 Results and discussion

### 3.1 Characteristics of CNC and zeolite particles

**FIGURE 1:**
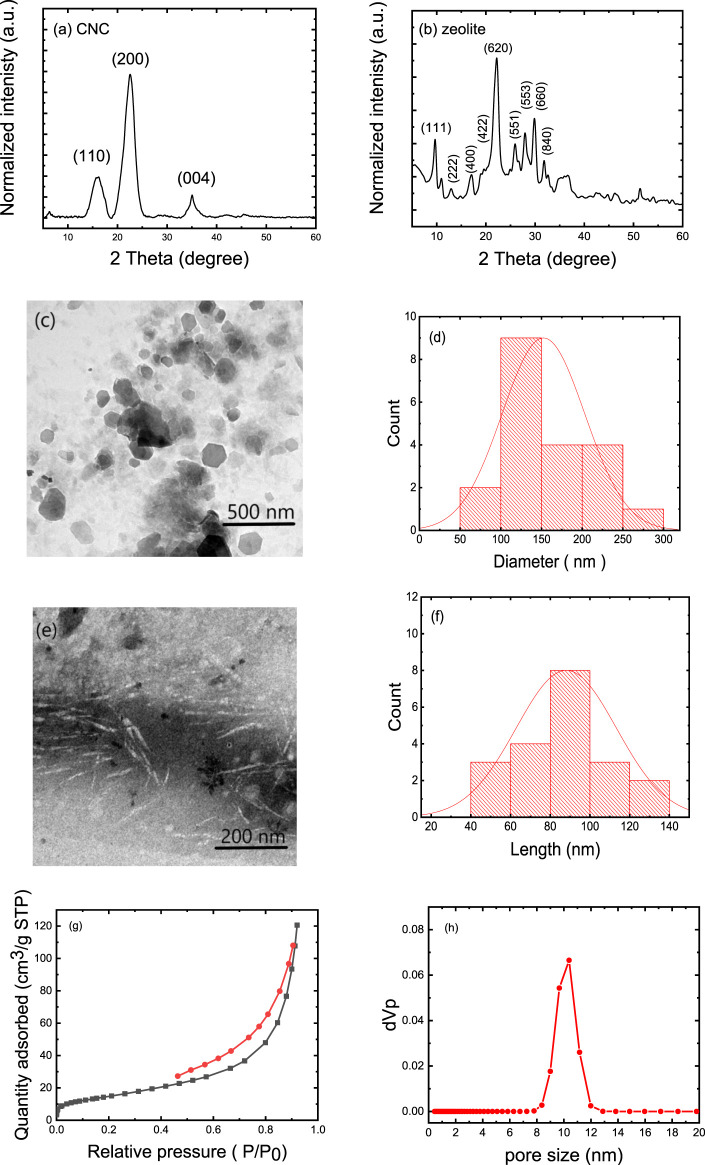
Characteristics of the zeolite and CNC: **(A)** XRD patterns of CNCs and **(B)** XRD patterns of zeolite; **(C)** TEM image of zeolite **(D)** Corresponding histogram of zeolite particle size distribution, **(E)** TEM image of CNC, **(F)** corresponding histogram showing the CNC particle length. **(G)** N_2_ adsorption-desorption isotherm, and **(H)** pore size distribution of zeolite particles.

The XRD patterns of CNC and zeolite are shown in Figures 1A, B, respectively. The CNC exhibited peaks at 2θ = 16.2°, 22.5°, and 35.0°, which are characteristic peaks for the cellulose and correspond to lattice planes (110), (200), and (004), respectively. The 2θ-peaks at 9°, 13°, 15°, 17°, 22°, 26°, 28°, 30°, 32°, and 36° are characteristic (111), (222), (400), (422), (620), (551), (553), (660) and (840) crystallographic planes of the crystalline zeolite (JCPDS:43-0168), which have already been shown in the zeolite diffractogram (Colombo, 2017). Figures 1C, D shows the TEM image and the corresponding particle size distribution of the zeolite particles, which are on average 150 ± 100 nm in size. Figure 1E shows the TEM image of the individual CNC and Figure 1F shows the corresponding particle size distribution of these particles. The TEM image shows that the CNC have a needle-like morphology with an average diameter of 10 ± 3 nm and a length of 90 ± 50 nm. The specific surface area and pore properties of natural zeolite (clinoptilolite) were evaluated volumetrically by the physisorption of N_2_ gas in static mode at normal boiling temperature (77 K) (Figures 1G, H). According to the pore size distribution results, zeolite particles have a maximum of about 10 nm. The zeolite particles have a BET surface area of 55.4 m^2^/g, and a total pore volume of 0.1865 cm^3^·g^−1^.

### 3.2 Membrane formation and MB dye adsorption mechanism

**FIGURE 2:**
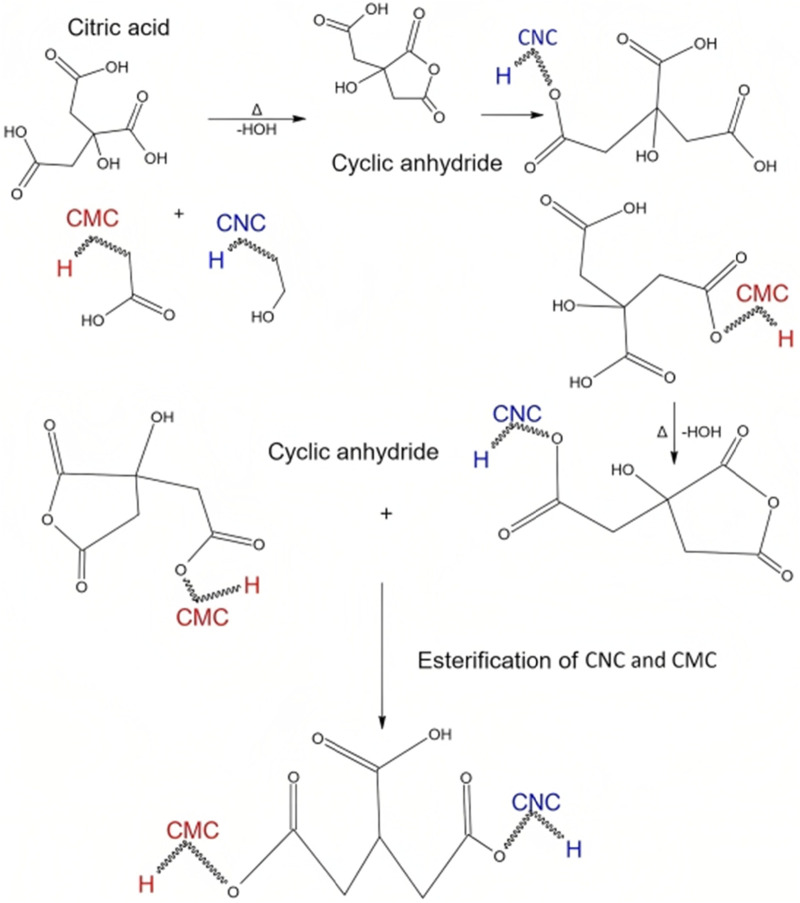
Hypothetical schematic representation of the formation of the cross-linked CNC/CMC/citric acid membrane.

Synthesis of CNCs undergoes a multi-step process, resulting in unique functional groups on the final product. Initial stages, involving dewaxing, demineralization, and alkalization, prioritize removing impurities and enhancing cellulose purity. Subsequent bleaching steps with H_2_O_2_ and NaOCl may introduce ester groups and the final acid hydrolysis with H_2_SO_4_ forms sulfate ester (-O-SO_3_H) and carboxyl (-COOH) groups on the CNC surface. The retention of hydroxyl groups (-OH) throughout the process contributes to the material’s hydrophilic nature. On the other side, the CMC is sodium salt from cellulose, which distinguishes itself from cellulose with anionic carboxymethyl groups (–CH_2_COONa) (Sharma et al., 2017b). The molecular weight (Mwt) and degree of substitution (DS) are crucial for CMC’s properties, and in this study, the DS was experimentally determined as 0.6. This CMC grade, retaining a significant number of OH groups, forms stable cross-links with CNC and citric acid, resulting in mechanically and dimensionally robust membranes in moist or aqueous conditions. Citric acid, chosen as the cross-linking agent for its non-toxic, cost-effective, and natural occurrence in fruits, can cross-link a maximum of two polymer chains (CMC/CNC). The crosslinking reaction may induce esterification, forming ester groups (-COOR) on both CNC and CMC, with carboxyl groups (-COOH) in CMC contributing to overall chemical modification. This crosslinking enhances stability and mechanical properties, making the resulting composite material suitable for diverse applications, such as biocompatible films or membranes (Kassem et al., 2020). During the 8-h drying phase at 60°C, further esterification of citric acid produces a cyclic anhydride, as depicted in Figure 2, illustrating limitations in tri-ester formation during the crosslinking reaction (Shen et al., 2015).

**FIGURE 3:**
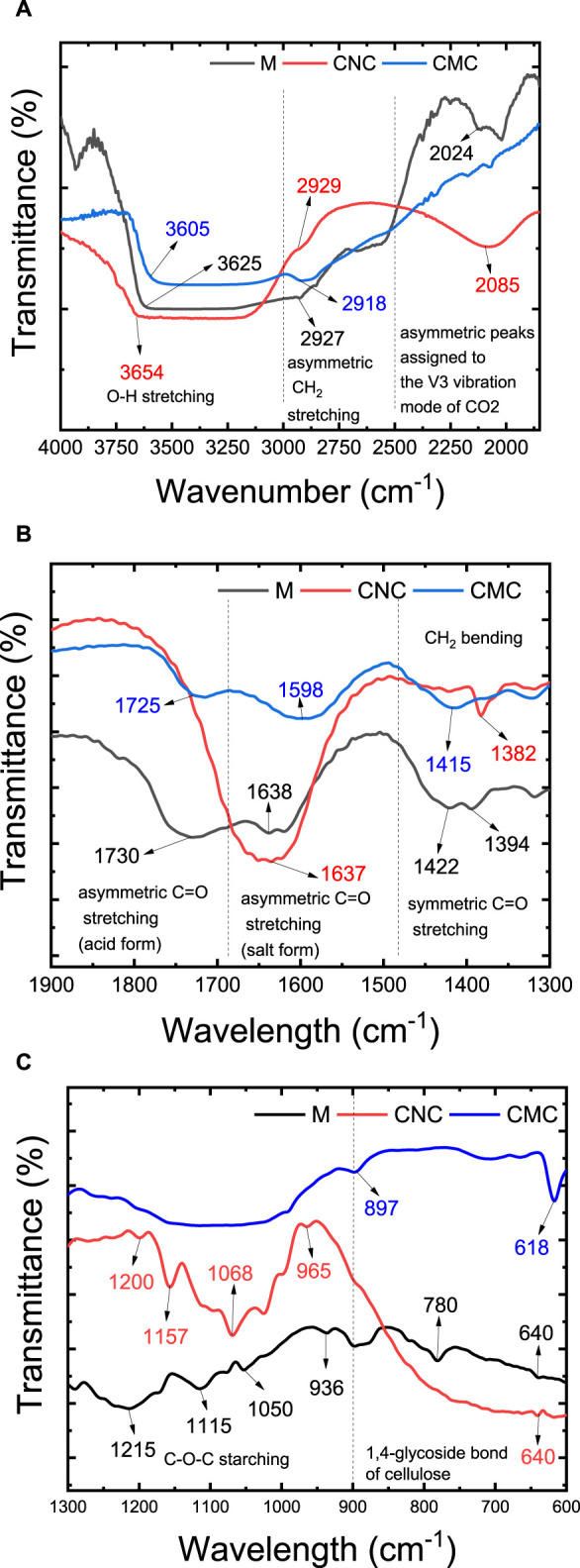
FTIR spectra of M1 membrane made of cross-linking of CMC, CNC, and citric acid without the addition of zeolite. **(A)** 4000–2000 cm^−1^; **(B)** 2000–1300 cm^−1^; **(C)** 1300–600 cm^−1^.

Alterations in functional groups resulting from the crosslinking reaction are contingent upon the specific conditions employed, and their characterization can be achieved through techniques such as FTIR spectroscopy and chemical analysis via SEM-EDX with elemental mapping. FTIR spectra are widely employed to validate the formation of ester linkages (Liang et al., 2020). In the study of the prepared membranes, the crosslinking reaction was investigated using FTIR, excluding the presence of zeolite (Figure 3). The IR spectrum of CMC showed the following characteristic peaks: O-H stretching (3,700–3,000 cm^−1^) and asymmetric CH_2_ stretching (3,000–2,800 cm^−1^). In the range of 2,300–2,000 cm^−1^, the very sharp and asymmetric peaks are assigned to the ν3 vibration mode of CO_2_ (Figure 3A). The strong absorption of C=O at 1,598 cm^−1^ confirms the asymmetric stretching vibration of carboxyl groups (COO-) and (1,415 cm^−1^) are assigned to the symmetrical vibration of carboxyl groups (COO–) as salts (Mondal et al., 2015). The C=O (acid form, -COOH) was also observed in the CMC sample at 1725 cm^−1^, which is assigned to the antisymmetric stretching vibration of C=O stretching, respectively (Capanema et al., 2018). The –CH_2_ bending or scissoring gives rise to a characteristic band near 1,450–1,470 cm^−1^ (Figure 3B). These bands may be overlapped with the band of adsorbed water. The strong vibrations that occur in the broad range of 950–1,250 cm^−1^, are attributed to the ether linkages (CH–O–CH_2_ stretching, see Figure 3C) in the CMC (Ghorpade et al., 2017; Ghorpade et al., 2018; Kassab et al., 2020). These peaks may be overlapped with C-O vibrations of -OH groups. The peak at 897 cm^−1^ is assigned to the 1,4-glycoside bond of cellulose (Figure 3C) (Mondal et al., 2015).

The IR spectrum of CNC showed four characteristic peaks including O–H stretching (3,658 cm^−1^), asymmetric –CH_2_ stretching (2,918 cm^−1^), –CH_2_ bending (1,382 cm^−1^), –OH bending vibration (1,330–1,320 cm^−1^), and C–O stretching (1,025 cm^−1^) (Gong et al., 2019; Kassab et al., 2019). The peaks at 1,600–1,620 cm^−1^ are attributed to carboxylate asymmetric stretching (COO−), which is formed because of primary -OH groups’ oxidation, induced by chemical treatment (i.e., acid treatment, alkali treatment, and bleaching) of bagasse. In the range of 2,600–2,000 cm^−1^, the very sharp and asymmetric peaks are assigned to the ν3 vibration mode of CO_2_. The peaks at 1,160 and 1,070 cm^−1^ are attributed to the saccharide structure. The IR peaks for sulfate ester groups include strong and sharp vibrations in the ranges of 1,200–1,300 cm^−1^ for sulfate stretching, 1,020 to 1,200 cm^−1^ for asymmetric stretching, and around 950–1,000 cm^−1^ for symmetric stretching. These distinctive peaks serve as indicators of the sulfate ester (-O-SO_3_H) functionalities in the CNC material.

As shown in the given spectra, after cross-linking of CNC, CMC, and citric acid, a peak shift was observed in the stretching C=O vibration of CMC (Demitri et al., 2008; Seki et al., 2014). This is verifying the effective crosslinking and formation of the ester group in the membrane matrix. The C=O stretch peak of CMC (1,725 cm^−1^ and 1,598 cm^−1^) are shifted to a higher wavelength (1,730 and 1,638 cm^−1^) and the bands at 1,115 cm^−1^ and 1,050 cm^−1^ are due to ether linkage between CMC and polycarboxylic acid (Park et al., 2015; Dacrory et al., 2018). Furthermore, the intensity of the absorption bands at 1,215–1,205 cm^−1^ increased, which was assigned to the vibration of formed C-O-C ester bonds (Wang et al., 2021). The inductive effect in the ester group altered the double bond character of the C=O group and causes the observed IR shift.

**FIGURE 4:**
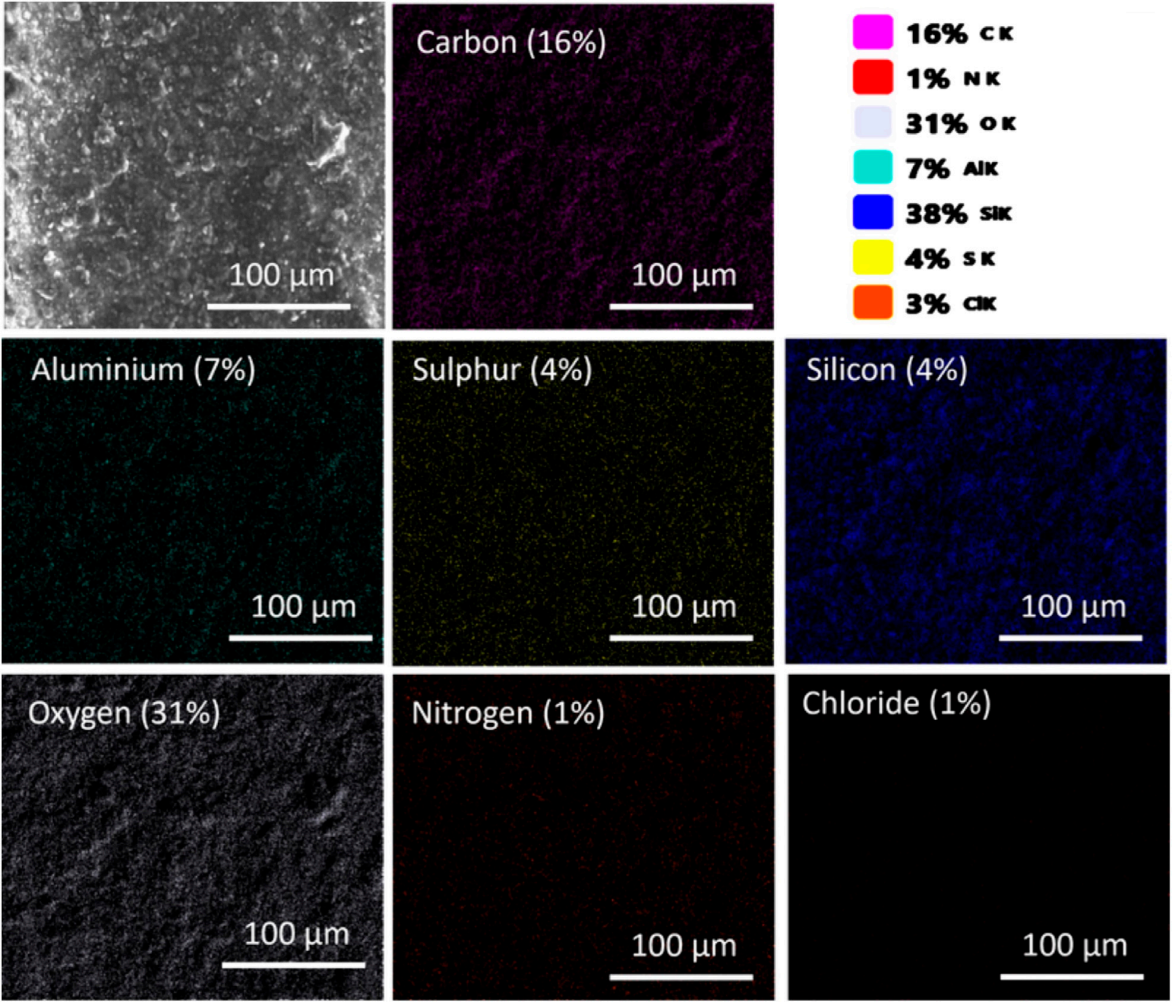
SEM-EDX analysis of the M5 membrane after MB dye adsorption. The M4 membrane consists of carbon and oxygen primarily from CNC and CMC, along with aluminum and silicon attributed to zeolite particles. Elemental distribution (Sulfur, Chlorine, and Nitrogen) corresponds to MB dye adsorption on the M5 membrane surface.

SEM microscopy assessed M5 membrane morphology (membrane composition in Table 1); EDS determined elemental composition post-MB dye adsorption. Optimized membrane effectiveness was confirmed under specified conditions (50 mg adsorbent mass, 50 ppm dye concentration, 50 mL solution, 120-min contact time, 25°C temperature). Aluminum and silicon from zeolite particles were consistently dispersed. The atomic composition of the M5 membrane before adsorption consisted of 10% Carbon (C), 28% Oxygen (O), 11% Aluminum (Al), and 51% Silicon (Si). After the adsorption of MB (Figure 4), the altered atomic composition of the M5 membrane included 16% Carbon (C), 31% Oxygen (O), 7% Aluminum (Al), 38% Silicon (Si), 1% Nitrogen (N), 4% Sulfur (S), and 3% Chlorine (Cl). Changes in elemental composition and the detection of sulfur (S), Chlorine (Cl), and Nitrogen (N) distribution are associated with MB dye, assisting in the identification of MB distribution within the membrane.

### 3.3 Swelling of the cross-linked membrane

**FIGURE 5:**
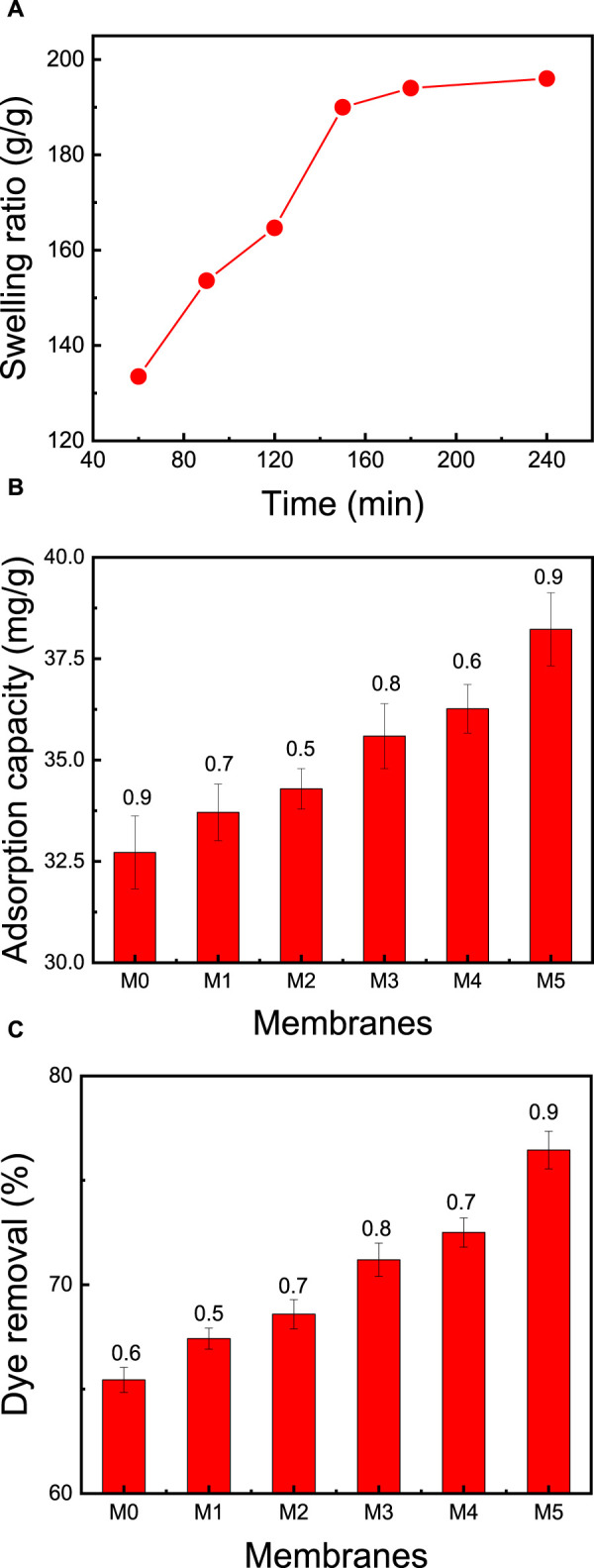
Effect of the membrane (CMC/CNC/zeolite/citric acid) composition on: **(A)** swelling ratio of the best membrane (M5) at neutral pH and ambient temperature of 25°C, **(B)** adsorption capacity, and **(C)** dye removal. The adsorption studies were conducted using a 50 mg membrane mass, 50 mL dye solution, an initial MB concentration of 50 ppm, solution pH = 7, room temperature of 25°C, and contact time of 2 h.

Swelling behavior in water is one of the most important properties of cellulose based membranes (Deng et al., 2021). Initially, water molecules diffuse slowly into the membrane, resulting in a swollen membrane. The swelling of the membrane over an area of 1 mm^2^ is called bulk swelling, while small dimensional changes are classified as microscopic swelling. CNC, CMC, and zeolite nanoparticles exhibit negative surface charges, which align with their high hydrophilicity. The membrane’s capacity to swell is contingent on factors like crosslink density, pH, and temperature. The degree of crosslinking, pore volumes, and the membrane’s functional attributes collectively dictate the extent of swelling it undergoes in specific liquids. Figure 5 illustrates a swelling degree of 134% for the membrane (CMC/CNC/zeolite/citric acid) after immersing it in distilled water for 60 min. Over the course of 60–120 min, the membrane’s swelling escalated from 134% to 164%. It eventually reached its maximum standard value of 194% after 160 min. This pronounced swelling is attributable to negatively charged zeolite particles and the presence of hydrophilic functional groups (e.g., OH, COOH) attached to the CNC and CMC chains. These factors enhance the membrane’s affinity for adsorbing cationically charged dyes from wastewater.

### 3.4 Mechanism of MB dye removal on the membrane

The adsorption mechanism of MB onto the membrane, composed of CMC, CNC, zeolite, and citric acid as a cross-linking agent, involves a multitude of interactions. The negatively charged surface of the membrane, attributed to carboxylate groups in CMC and CNC, engages in electrostatic interactions with the positively charged MB molecules. The significant presence of hydroxyl (-OH), sulfate ester (-O-SO_3_H), and carboxyl (-COOH) groups in CNC plays a crucial role in facilitating MB adsorption. Hydrogen bonding further enhances this process between the hydroxyl groups in cellulose-based materials and MB dye. The collective interplay of electrostatic interactions, ion exchange, hydrogen bonding, chemical binding, pore filling, and considerations of surface area collectively contribute to the membrane’s efficacy in adsorbing MB dye.

The porous structure and high surface area of zeolite facilitate both pore filling and surface adsorption of MB dye, with its ion exchange capacity enabling cation exchange with the dye molecules. Zeolite emerges as an economical and environmentally friendly adsorbent for cationic dyes, releasing non-toxic exchangeable ions (e.g., Ca^2+^, Mg^2+^, K^+^, Na^+^) into the water system. With MB dye molecules having dimensions of approximately 14 Å in length and 9.5 Å in width, zeolites, with pores of less than 2 nm, are ideally suited for entrapping MB dye molecules. CMC’s efficacy in enhancing adsorption capabilities has been validated in previous research, such as Li et al. (2020)’s work, where a CMC/CNC-based hydrogel achieved maximum adsorption capacity under specific pH conditions and contact time.

The incorporation of citric acid as a cross-linking agent not only enhances membrane structural integrity through chemical bonding but also allows for potential complex formation between citric acid and metal ions in MB dye molecule, influencing adsorption behaviour. Additionally, Van der Waals forces are presumed to contribute to interactions between CNC and zeolite components within the membrane. Subsequent sections will delve into adsorption studies to explore the specific interactions within this complex adsorption system.

### 3.5 Effect of membrane composition on dye removal

Adsorption serves as a commonly employed technique for the treatment of wastewater contaminated with dyes. The primary challenge in sustainable adsorbents is achieving a substantial dye adsorption capacity. In pursuit of a high dye removal capacity, six distinct membranes were fabricated, each featuring varying concentrations of CNC, CMC, zeolite, and citric acid, as detailed in Table 1. These six membranes were subjected to testing using a consistent concentration of MB dye (50 mg/L) within a 50 mL dye solution, initiated at a pH of 7 and a temperature of 25°C.

Upon elevating the CNC content in M0 and M1 from 0% to 75%, and concurrently reducing the zeolite content from 20% to 0%, there was a marginal increase in the adsorption capacity, rising from 65.0% to 67.4%. This observation suggests that augmenting the CNC:CMC ratio has a discernible effect on the removal of MB dye (as portrayed in Figure 5). The rationale behind this could be attributed to the presence of negatively charged groups arising from increased ratios of both CMC and CNC, which ultimately augment the dye adsorption process. The inclusion of zeolite particles and optimization of membrane composition further contribute to the enhancement of MB dye removal. Notably, the most effective membrane, M5, exhibited a dye removal rate of 76.5% and a remarkable dye adsorption capacity of 38.2 mg/g. This performance was achieved at an initial MB concentration of 50 ppm, an initial pH of 7, a temperature of 25°C, and a contact time of 2 h.

### 3.6 Effect of initial MB dye concentration on dye removal

**FIGURE 6:**
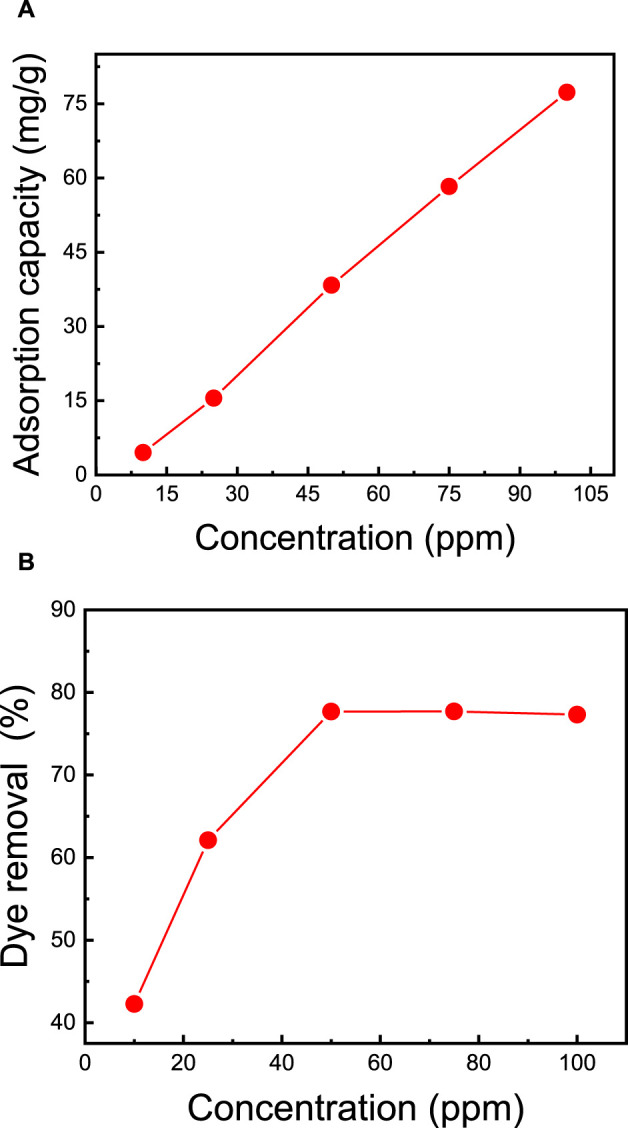
Effect of the initial MB dye concentration on **(A)** adsorption capacity and **(B)** dye removal. The adsorption studies were conducted using the M5 membrane with mass of 50 mg, 50 mL dye solution, initial pH = 7, room temperature of 25°C, and contact time of 2 h.

The effectiveness of dye removal from water is significantly influenced by the initial concentration of methylene blue (MB) dye and the mass of the membrane. A higher membrane mass provides an increased number of sorption sites, correlating with enhanced MB dye removal efficiency (Rahman et al., 2022). In this study, additional MB adsorption experiments were conducted using the top-performing membrane, M5, covering a range of initial MB dye concentrations from 10 ppm to 100 ppm. Each dye solution had a consistent volume of 50 mL, the membrane mass was maintained at 50 mg, the pH was set at 7, and stirring occurred at 400 rpm for 2 h. Figure 6 illustrates the gradual increase in adsorption capacity from 4.5 mg/g to 78.0 mg/g as the MB dye concentration increases from 10 ppm to 50 ppm, resulting in an increase in removal from 42.3% to 77.7%. However, further elevating the dye concentration from 50 ppm to 100 ppm does not yield any enhancement in adsorption capacity. The observed trend in the adsorption curves reveals a direct correlation between MB concentration and both adsorption capacity and removal efficiency. This underscores the nanocellulose-based membrane’s efficacy in handling elevated MB concentrations, providing crucial information for optimizing its performance under varying dye concentrations.

### 3.7 Effect of contact time on MB dye removal

**FIGURE 7:**
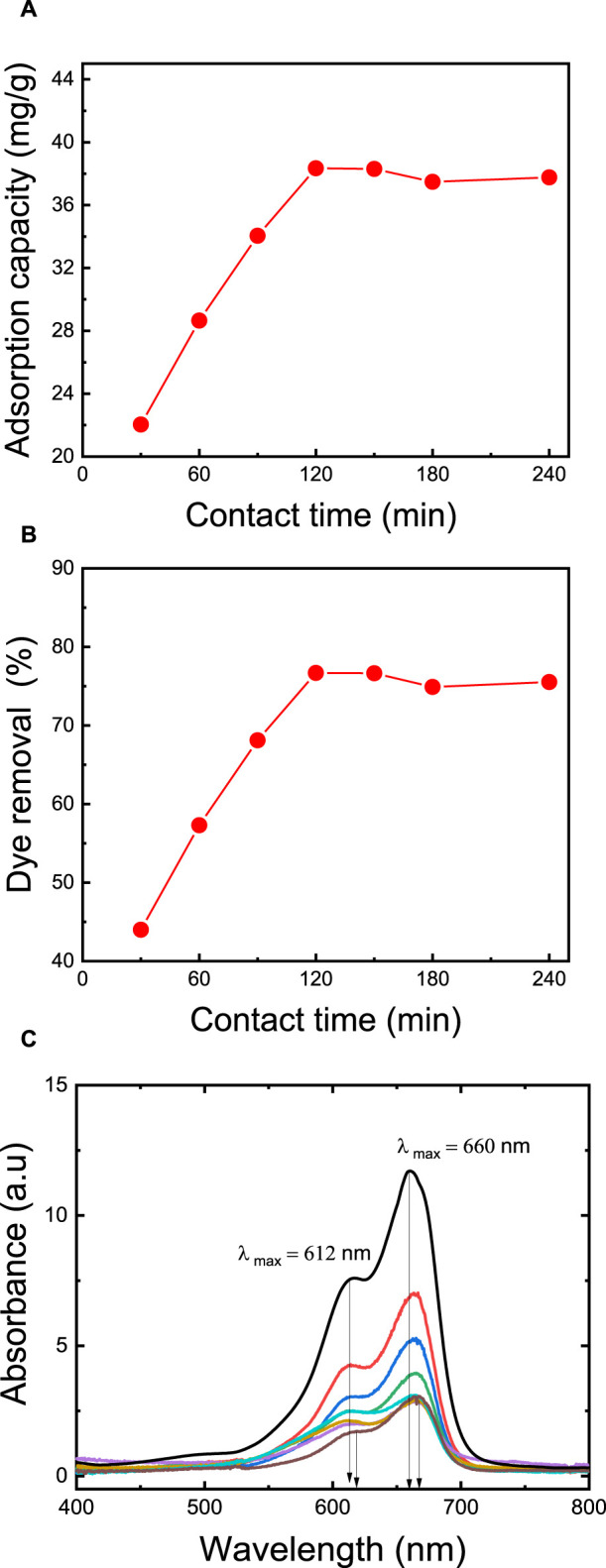
Effect of contact time on: **(A)** adsorption capacity, **(B)** dye removal percentage, **(C)** the kinetics of adsorption thoroughly via a full-scan mode of UV-vis spectroscopy. The adsorption studies were conducted using the M5 membrane with mass of 50 mg, 50 mL dye solution, an initial MB concentration of 50 ppm, an initial pH = 7, and a temperature of 25°C.

Dye removal is positively influenced by an extended contact time, primarily because it enhances the likelihood of dye molecules binding to the available adsorption sites on the membrane. The duration required to achieve equilibrium signifies the membrane’s maximum adsorption capacity. To explore the impact of contact time, a series of adsorption experiments were carried out at varying time intervals, ranging from 30 to 240 min. The results revealed a gradual increase in the quantity of adsorbed MB dye with longer contact times (as depicted in Figure 7). During the initial 120 min, the adsorption capacity rises with time until equilibrium is attained (Abou Oualid et al., 2020). By extending the contact time from 30 to 120 min, the adsorption capacity increases from 22.0 mg/g to 37.8 mg/g, and the dye removal rate rises from 44.0% to 76.0%. These findings align with the results of Somsesta et al. (2020), who studied the impact of exposure time in their research involving activated carbon/cellulose composite films for MB dye removal. Their study revealed that adsorption capacity increased with contact time until equilibrium was reached. About 70% adsorption was achieved within 360 min, with equilibrium taking 24 h to stabilize. The maximum MB adsorption capacity was recorded at 103 mg/g at a dye concentration of 100 ppm, an initial pH of 6.9, and a temperature of 35°C for 24 h. In-depth analysis of the adsorption kinetics was performed using UV-Vis spectroscopy in full-scan mode (Figure 7C), wherein the observed redshift (bathochromic shift) of 7.5 nm in the UV-Vis spectrum, transitioning from 0 min (*λ* max = 660 nm) to 240 min (*λ* max = 667.5 nm), signifies the strengthening of ionic interactions between MB and the membrane (Karmakar et al., 2017).

### 3.8 Effect of temperature of MB dye removal

**FIGURE 8:**
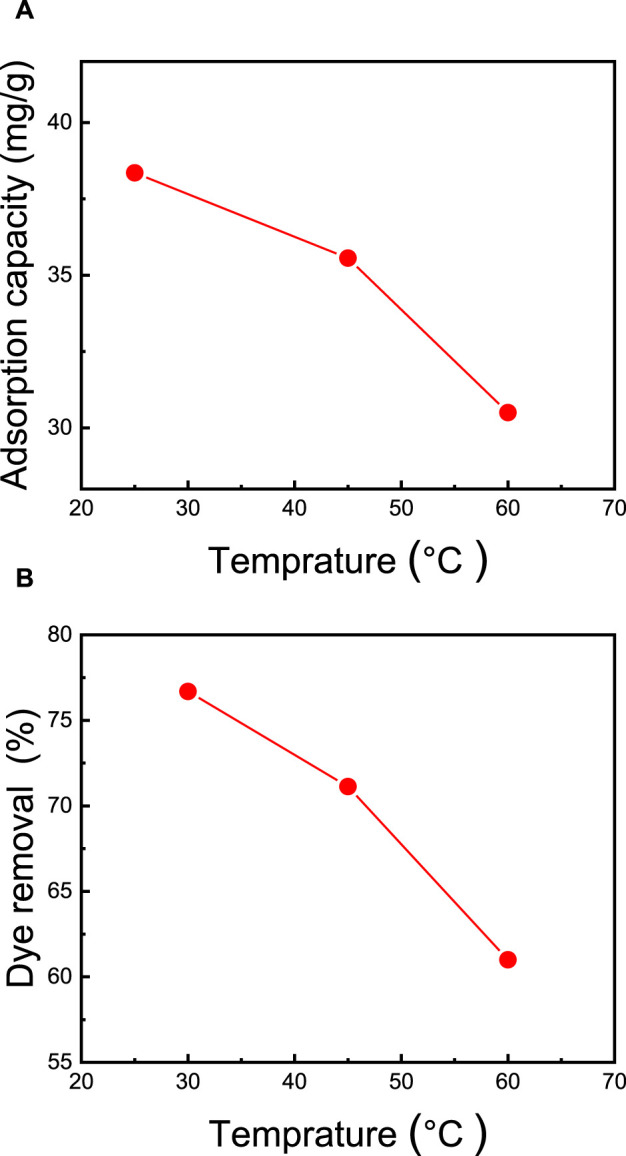
Effect of temperature on **(A)** adsorption capacity and **(B)** percentage of dye removal. The adsorption studies were conducted using the M5 membrane with mass of 50 mg, 50 mL of dye solution, an initial MB concentration of 50 ppm, an initial pH = 7, and a contact time of 2 h.

The temperature of the dye solution stands out as a critical factor significantly impacting adsorption capacity. In general, in physical adsorption, there exists an inverse relationship between adsorption capacity and temperature. To explore the temperature’s influence, adsorption experiments were conducted at various temperatures, specifically 25°C, 40°C, and 60°C. As illustrated in Figure 8, it becomes evident that the adsorption of MB dye diminishes as the temperature increases, decreasing from 38.4 mg/g to 30.5 mg/g, while the dye removal rate decreases from 77.0% to 61.0% (as depicted in Figure 8). This decline in MB dye removal at elevated temperatures can be ascribed to a weakening of the adsorption forces between the MB molecules and the active sites within the membrane. It can also be attributed to interactions between neighboring adsorbed phase molecules (Senthil Kumar et al., 2014). These findings are in concurrence with the results reported by Mohammed et al. (2015), who examined CNC/alginate-based hydrogel beads and evaluated the impact of temperature on the removal of MB dye. In their investigation, they observed that raising the temperature from 25°C to 55°C led to a reduction in dye removal efficiency, dropping from 77% to 69%.

### 3.9 Effect of pH and pHpzc on MB removal

The pH of the dye solution plays a pivotal role in regulating the extent of electrostatic charges transferred from ionized dye molecules, which subsequently impacts dye adsorption capacity (Steinfeld et al., 2015). Specifically, the adsorption of cationic dyes is more favorable at higher pH levels, whereas the efficiency of anionic dye adsorption is relatively lower. This phenomenon is attributed to the increase of surface charge density with increasing pH (2–12). The consequence is a strengthened electrostatic attraction between the positively charged dye (MB) and the negatively charged membrane surface, resulting in an increased adsorption capacity (Coşkun et al., 2021). The alteration in pH levels exerts an influence on both the ionization of functional groups on the membrane surface and the ionization of MB dye molecules.

**FIGURE 9:**
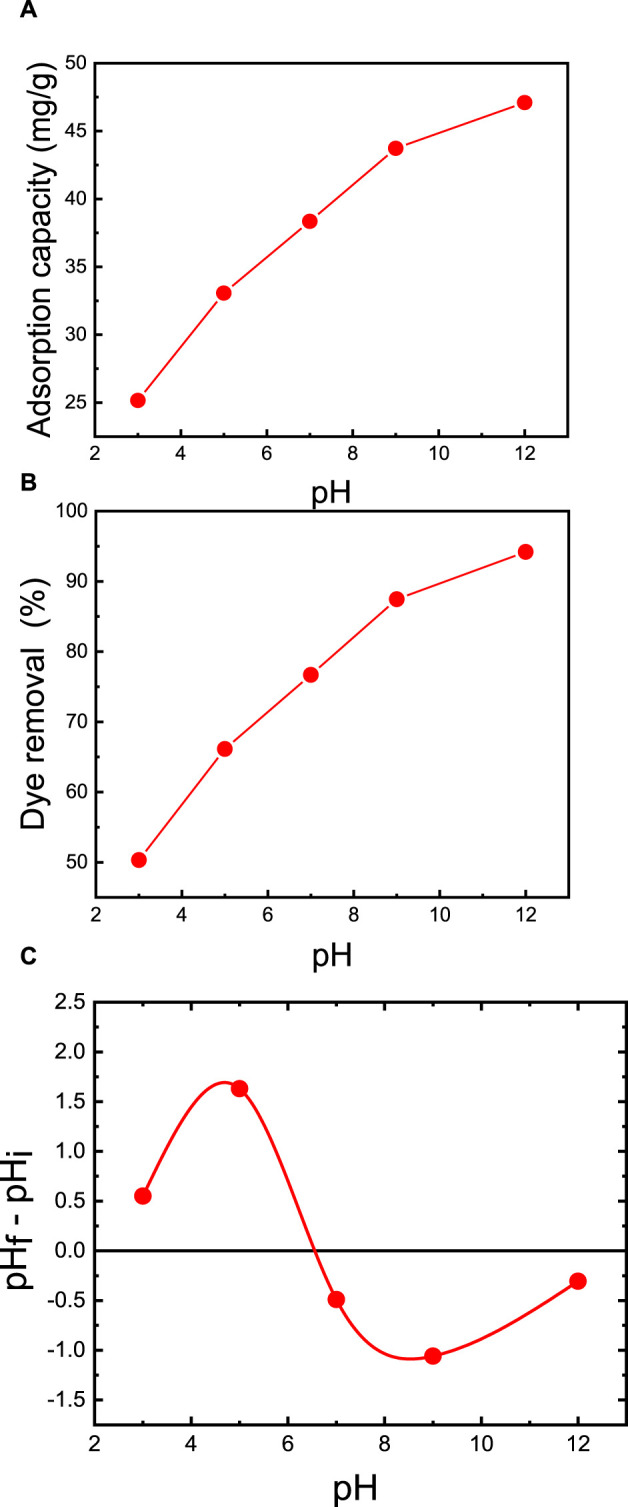
Effect of initial pH on MB dye removal of the M5 membrane: **(A)** adsorption capacity and **(B)** percent dye removal. **(C)** Effect of point of zero charges (pHpzc) on adsorption capacity of MB. The adsorption studies were conducted using the M5 membrane with mass of 50 mg, 50 mL of dye solution, an initial MB concentration of 50 ppm, a room temperature of 25°C, and a contact time of 2 h.

To examine the effect of pH, experiments were conducted under consistent conditions, with the best membrane (M5), membrane support mass of 50 mg, 50 mL of dye solution, an initial MB concentration of 50 ppm, and a temperature of 25°C. The solution was adjusted to varying initial pH values of 3, 5, 7, 9, and 12, using dilute HCl and NaOH. As presented in Figure 9, at a pH of 3, the adsorption capacity is 25.2 mg/g, with a removal efficiency of 50.3%. In contrast, at pH 7, the dye removal significantly increases to 76.7%. Further elevating the initial pH from 7 to 12 yields an exceptional dye removal efficiency of 94.2% and an adsorption capacity of 47.1 mg/g. Consequently, pH values above 8 prove advantageous for MB adsorption on the prepared membrane. These findings concur with those reported by Oyewo et al. (2020), who developed an adsorbent based on CNC/ZnO-NPs and examined the impact of initial pH. Their research revealed that adjusting the initial pH from 2 to 10 resulted in enhanced dye removal, increasing from 60% to 90% (Oyewo et al., 2020).

The zero point of charge (pHpzc) emerges as a pivotal factor affecting the surface adsorption capacity of the membrane. Owing to the presence of functional groups such as OH- and COO- groups on the membrane, the adsorption of the cationic MB dye is favored when the pH is above the pHpzc. The pHpzc signifies the point at which the curve representing pH = (pH-final - pH-initial as a function of pH-initial) intersects the abscissa axis. As showcased in Figure 9C, the M5 membrane possesses a pHpzc of 6.5. This implies that the adsorbent’s surface carries a positive charge when the pH is below 6.5 and a negative charge when the pH exceeds 6.5. As the pH approaches the pHpzc, the negative ion density on the M5 membrane’s surface increases, intensifying the adsorption of MB dye. This is further supported by the diminished binding of MB dye molecules in strongly acidic conditions (pH = 2), primarily due to the repulsion among MB dye molecules, leading to a decline in dye adsorption. The highest MB dye adsorption on M5 occurs above the pHpzc (pH = 6.5) because the number of negative sites prevails, resulting in a more robust electrostatic attraction between MB dye and the membrane (Ayouch et al., 2020; Chen and Wang, 2007). These outcomes align with adsorption experiments illustrating that the MZ membrane in an alkaline solution (pH 8–12) exhibits exceptional adsorption, increasing from 80% at natural pH to 94%.

### 3.10 Adsorption kinetics and thermodynamic isotherm

**FIGURE 10:**
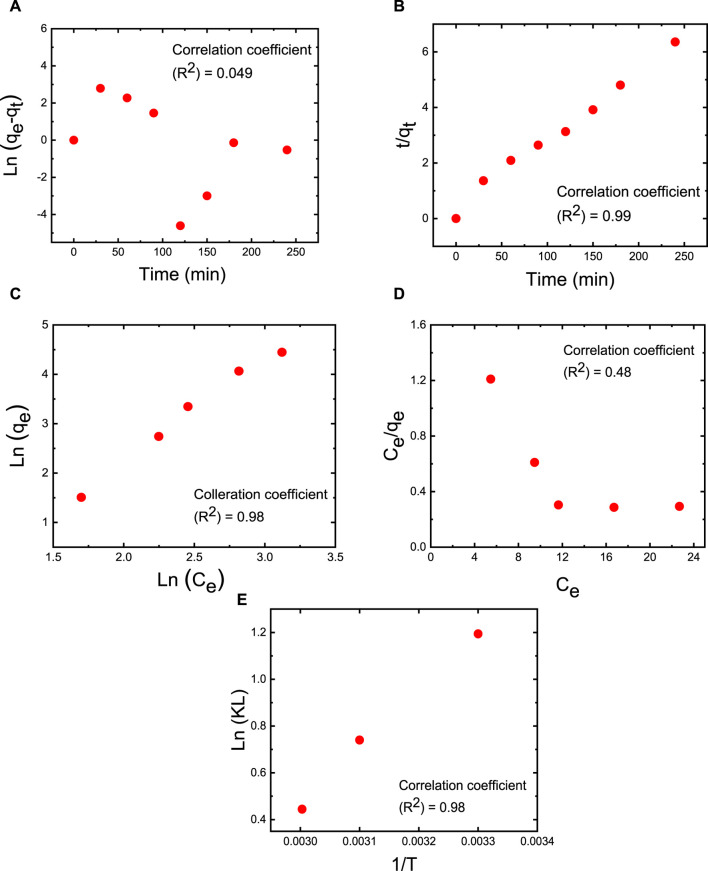
Adsorption experiments of MB dye on M5 membrane using linear fitting with **(A)** pseudo-first-order kinetics at an operating temperature of 25°C; **(B)** pseudo-second-order kinetics at an operating temperature of 25°C, **(C)** Freundlich isotherm at 25°C (298 K), 40°C (318 K) and 60°C (333 K), **(D)** Langmuir isotherm at 25°C (298 K), 40°C (318 K) and 60°C (333 K). **(E)** Impact of temperature on MB dye adsorption conducted at temperatures of 25°C (298 K), 40°C (318 K), and 60°C (333 K). The adsorption studies were conducted using the M5 membrane with mass of 50 mg, 50 mL dye solution, an initial MB concentration of 50 ppm, an initial pH = 7, and a contact time of 2 h.

Adsorption kinetics was employed to evaluate the capacity of MB dye to adsorb over time while maintaining a constant dye concentration (Onu et al., 2023). These kinetic experiments were conducted using an M5 membrane with a mass of 50 mg, a 50 mL dye solution with an initial MB concentration of 50 ppm, an initial pH of 7, a temperature of 25°C, and a contact time of 2 h (Figure 10). The experimental determination of the adsorption capacity yielded approximately 76.7 mg/g. To analyze the experimental kinetic data, the pseudo-first-order and pseudo-second-order kinetic models were employed and fitted to the data (as depicted in Figures 10A, B). The adsorption capacity at equilibrium time (qe = 40.2 mg/g), the correlation coefficient (*R* ^2^ = 0.986), and the reaction constant (k2 = 0.001821 g/mg·min) obtained with the pseudo-second-order model closely align with results from other models in the literature. In contrast, the adsorption capacity at equilibrium time (qe = 3.546 mg/g), the correlation coefficient (*R* ^2^ = 0.049), and the reaction constant (k1 = 0.014 min^−1^) derived from the pseudo-first-order model significantly deviate from results obtained in other studies. This suggests that the adsorption process of the investigated adsorbates is predominantly driven by chemisorption, and the pseudo-second-order mechanism prevails in the MB adsorption process (Chen and Wang, 2007).

Thermodynamic adsorption isotherms were established by fitting the experimental data collected at the equilibrium time point with Freundlich and Langmuir isotherm models (Figures 10C, D, respectively). These models describe the relationship between adsorption capacity and equilibrium concentration under constant temperature conditions (Sharma et al., 2018b). According to the Langmuir adsorption model, a saturated monolayer of dye molecules on the membrane’s surface represents the maximum limited absorption capacity (Singha et al., 2017). In contrast, the Freundlich adsorption isotherm suggests that adsorption on a heterogeneous surface occurs through a multilayer adsorption mechanism, and the adsorbed amount increases with an augmentation in MB dye concentration (Sunderland et al., 2011). The linear forms of these isothermal models are expressed in Eqs 10, 11.

$$\frac{C_{e}}{q_{e}}=\frac{K_{s}}{q_{\max}}+\frac{C_{e}}{q_{\max}}$$ (10)

$$\log q_{e}=\frac{1}{n}\log C_{e}+\log p$$ (11)

(Sunderland et al., 2011) In the equations, qe represents the amount of MB adsorbed at equilibrium (in mg/g), Ce is the equilibrium concentration of the dye in the solution (in mg/L), qm signifies the maximum adsorption capacity of the membrane with a given mass, while kL and kF are the constants for the Langmuir and Freundlich models, respectively. Based on the correlation coefficient (R2) values, the Freundlich isotherm stands out as the most suitable model for characterizing the MB dye adsorption process (as illustrated in Figure 10C). The favorable fit of the Freundlich isotherm indicates that MB molecules form a multilayer adsorption on the heterogeneous membrane sites, effectively covering the membrane’s surface. As indicated in Figure 10E, experiments examining the impact of temperature on MB dye adsorption were conducted at temperatures of 25°C (298 K), 40°C (318 K), and 60°C (333 K). Thermodynamic parameters, including ΔS, ΔH, and ΔG, were determined based on the adsorption capacity (qe in mg/g) and concentration (Ce in mol·L^−1^). The outcomes reveal that as the temperature increases from 25°C (298 K) to 40°C (318 K) and further to 60°C (333 K), ΔG becomes less negative (transitioning from −2.95 kJ·mol^−1^ to −2.38 kJ·mol^−1^ and then to −1.23 kJ·mol^−1^, respectively). This suggests that higher temperatures are not conducive to the spontaneous adsorption of MB, aligning with the results presented in Figure 8. The ΔH value for MB adsorption is −1.72 kJ·mol^−1^, indicating that the adsorption of MB molecules onto the M5 membrane represents an exothermic physical adsorption process. Additionally, the negative ΔS values of −47.42 kJ.mol^−1^·K^−1^ suggest that the randomness at the interface between the membrane and the dye solution decreases during the adsorption process (Alkan et al., 2007). Finally, the activation energy of adsorption (Ea) was assessed using an Arrhenius-type equation, as shown in Eq 12.

$$k2=¼\;k0\exp\left[\text{Ea}/\text{RT}\right]$$ (12)

(Alkan et al., 2007) Estimated Ea values based on the slope of the linear plot of ln k2 vs. 1/T where, k^0^, R, and T are the temperature independent factor (g·mg^−1^·min^−1^), the universal gas constant (8.314 J·mol^−1^·K^−1^) and the solution temperature (K), respectively = −16.72 J·mol^−1^·K^−1^ (Singha et al., 2017).

Adsorption capacity is the amount of MB dye taken up by the prepared membrane per unit mass. Compared to other adsorbents, the best membrane (M5) has higher adsorption capacities for MB absorption, as shown in Table 2. The membrane’s composition, comprising CMC, CNC, zeolite, and citric acid as a cross-linking agent, demonstrates a remarkable dye adsorption capacity of 38.2 mg/g. This surpasses the capabilities of other natural adsorption materials, such as Moroccan cactus (3.4 mg/g), dried cactus (14.0 mg/g), sugar scum (24.5 mg/g), microcrystalline cellulose (12.9 mg/g), fly ash (1.3 mg/g), and zeolite alone (12.7 mg/g). The significant enhancement in adsorption capacity exhibited by the CMC/CNC/zeolite/citric acid membrane underscores its effectiveness in comparison to these natural adsorbents, emphasizing its potential for advanced applications in MB dye removal from wastewater. The comprehensive approach to membrane composition and design in this work contributes to its superior performance, marking a noteworthy advancement in sustainable water treatment solutions.

**TABLE 2:** Difference examples of natural adsorption used for MB dye removal compared to the best membrane (M5) reported in this work.

Membrane composition	Dye adsorption capacity	References
Moroccan cactus	3.4 mg/g	Sakr et al. (2020)
Dried Cactus	14.0 mg/g	Sakr et al. (2020)
Sugar Scum	24.5 mg/g	El Maguana et al. (2018)
Microcrystalline cellulose	12.9 mg/g	Tan et al. (2018)
Fly ash	1.3 mg/g	Woolard et al. (2002)
Zeolite	12.7 mg/g	Woolard et al. (2002)
Cellulose nanofibers reinforced alginate-polyvinyl alcohol hydrogels	36.3 mg/g	Yue et al. (2016)
Humic acid immobilized on a polypropylene supported sodium alginate/hydroxyethyl cellulose blend membrane	15.39 mg/g	Shenvi et al. (2015)
CMC/CNC/zeolite/citiric acid	38.2 mg/g	This work

## 4 Conclusion

This study highlights the potential applications of cellulose nanocrystals (CNCs), carboxymethyl-cellulose (CMC), zeolites, and citric acid in developing highly effective nanocellulose-based membranes to remove methylene blue (MB) dye from wastewater. The use of CNCs from agricultural waste like sugarcane bagasse, combined with eco-friendly crosslinking agent (citric acid), showcases the environmentally conscious approach of this membrane design. Following systematic experimentation, the optimal membrane composition is determined as 60% CNC, 15% CMC, 20% zeolites, and 5% citric acid, achieving a 79.9% dye removal efficiency and a 38.3 mg/g adsorption capacity at pH 7. Under specified conditions (50 mg adsorbent mass, 50 ppm dye concentration, 50 mL solution, 120-min contact time, and 25°C temperature), the optimized membrane proves effective. Increasing pH from 8 to 12 enhances MB dye removal efficiency from 79.9% to 94.5%, with the adsorption capacity rising from 38.3 mg/g to 76.5 mg/g. This efficiency is attributed to the membrane’s surface acquiring a highly negative charge beyond pH 8, facilitating electrostatic attraction forces for MB dye adsorption. Chemical thermodynamic experiments identify the Freundlich isotherm as the most accurate model, revealing multiple layers of MB dye adhering to heterogeneous negative sites on the membrane. Chemical kinetic experiments reveal pseudo-second-order kinetics, indicating a chemisorption mechanism. Utilizing the Langmuir isotherm model to elucidate a monolayer adsorption process and the Freundlich isotherm model to provide insights into multilayer adsorption of MB dye and in optimizing the adsorption mechanisms of MB dye on the membrane surface. The results contribute valuable insights for meaningful comparisons with other studies, supporting ongoing efforts for sustainable water treatment and environmental preservation.

## Data Availability

The raw data supporting the conclusion of this article will be made available by the authors, without undue reservation.
